# Evaluation of Dermal Substitute in a Novel Co-Transplantation Model with Autologous Epidermal Sheet

**DOI:** 10.1371/journal.pone.0049448

**Published:** 2012-11-07

**Authors:** Guofeng Huang, Shizhao Ji, Pengfei Luo, Yunqing Zhang, Guangyi Wang, Shihui Zhu, Shichu Xiao, Zhaofan Xia

**Affiliations:** Burns Institute of the PLA, Affiliated Changhai Hospital of the Second Military Medical University, Shanghai, China; University of California Merced, United States of America

## Abstract

The development of more and more new dermal substitutes requires a reliable and effective animal model to evaluate their safety and efficacy. In this study we constructed a novel animal model using co-transplantation of autologous epidermal sheets with dermal substitutes to repair full-thickness skin defects. Autologous epidermal sheets were obtained by digesting the basement membrane (BM) and dermal components from rat split-thickness skins in Dispase II solution (1.2 u/ml) at 4°C for 8, 10 and 12 h. H&E, immunohistochemical and live/dead staining showed that the epidermal sheet preserved an intact epidermis without any BM or dermal components, and a high percentage of viable cells (92.10±4.19%) and P63 positive cells (67.43±4.21%) under an optimized condition. Porcine acellular dermal matrixes were co-transplanted with the autologous epidermal sheets to repair full-thickness skin defects in Sprague-Dawley rats. The epidermal sheets survived and completely re-covered the wounds within 3 weeks. Histological staining showed that the newly formed stratified epidermis attached directly onto the dermal matrix. Inflammatory cell infiltration and vascularization of the dermal matrix were not significantly different from those in the subcutaneous implantation model. Collagen IV and laminin distributed continuously at the epidermis and dermal matrix junction 4 weeks after transplantation. Transmission electron microscopy further confirmed the presence of continuous lamina densa and hemidesmosome structures. This novel animal model can be used not only to observe the biocompatibility of dermal substitutes, but also to evaluate their effects on new epidermis and BM formation. Therefore, it is a simple and reliable model for evaluating the safety and efficacy of dermal substitutes.

## Introduction

The development of favorable dermal substitutes has been a major focus in skin tissue engineering research [1]. Dermal substitutes can serve as the structural template for wound healing by inducing dermal reconstruction, regulating the proliferation and differentiation of keratinocytes, and promoting the formation of an intact and functional basement membrane (BM) [2]. Large numbers of new dermal substitutes have already been derived either from natural materials such as the acellular dermis or from artificial materials such as collagen, hyaluronic acid hydrogel and electrospun nanomaterials [3]–[7], and many other studies are also in progress.

The development of new dermal substitutes requires a reliable and effective animal model to evaluate their safety and efficacy, including biocompatibility, immunogenicity, vascularization, and their ability to reconstruct dermal structure and promote new epidermis and BM formation [8]–[10]. Models currently available for evaluating dermal substitutes include the model of constructing composite skin substitutes in vitro, the subcutaneous implantation model, and the wound healing model [10]–[16]. The in vitro model plays an important role in early elimination of unsuitable dermal substitutes. But as the in vitro behaviors of keratinocytes and fibroblasts are quite different from their in vivo pattern, and the dermal substitute will undergo gradual degradation in the body, this model cannot replace the process of in vivo experiments. The subcutaneous implantation model is mainly used to investigate the biocompatibility and degradation pattern of dermal substitutes, but it is unable to directly evaluate the effects of dermal substitutes on new epidermis formation and dermal reconstruction. The wound healing model is the most reliable model as it can replicate clinical conditions. However, problems also exist, because the surgical procedures used in each study are extremely different and the results obtained are quite different in each model.

In this study, we have constructed a novel animal model to evaluate dermal substitutes. The rat split-thickness skin was harvested and treated with Dispase II solution to obtain an intact epidermal sheet that preserved high cell viability and proliferating ability, and then the autologous epidermal sheet was co-transplanted with porcine acellular dermal matrix (ADM) to repair full-thickness skin defect. This novel animal model is easy to follow and can be used to directly observe the effects of dermal substitutes on new epidermis and BM formation. It may prove to be a reliable model for evaluating the safety and efficacy of dermal substitutes.

## Materials and Methods

### Materials

This research protocol was approved by the Committee on the Ethics of Animal Experiments of the Second Military Medical University (Shanghai, China) and all animal experiments were performed in strict accordance with the NIH Animal Care & Use Guidelines. Dispase II was purchased from Sigma Aldrich (St. Louis, MO, USA). Cell counting kit 8 (CCK-8) and Hoechst 33342/Propidium Iodide (Hoe/PI) assay kit were supplied by Beyotime (Beijing, China). Sprague Dawley (SD) rats were from *B&K Universal Group* (Shanghai, China). All the other chemicals were of reagent grade and used as received without further purification.

**Figure 1:**
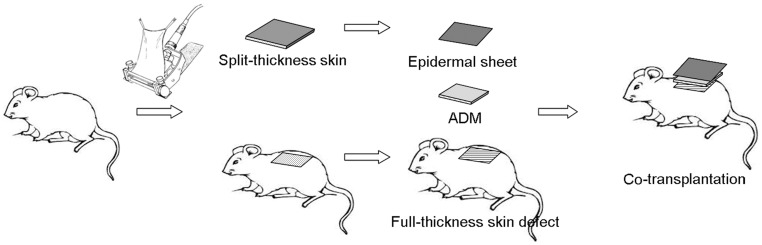
Surgical procedures of co-transplantation. The autologous epidermal sheet and ADM are co-transplanted to the full-thickness skin defect.

### Preparation of Epidermal Sheet

SD rats of clean grade weighing 160–180 g were anesthetized by intraperitoneal injection of 1% sodium pentobarbital. After the back was shaven and sterilized with iodophor, a split-thickness skin measuring 3×3 cm (0.3–0.5 mm in thickness) was harvested with a Zimmer skin graft blade (Zimmer Inc, IN, USA). The split-thickness skin was immersed in Dispase II solution (1.2 U/ml in phosphate buffered saline(PBS)) and incubated at 4°C with agitation for 8, 10 and 12 h, and then rinsed thoroughly with PBS. The epidermal sheet was separated bluntly from the dermis by forceps and placed in PBS for use.

### Histological Observation of Epidermal Sheet

The epidermal sheet specimens were fixed in 4% paraformaldehyde, dehydrated with an increasing series of alcohol concentrations, paraffin embedded and sliced into 5 µm sections. The sections were then deparaffinized and stained with hematoxylin and eosin (H&E), and finally examined under a light microscopy (Nikon Eclipse 80i, Japan). The split-thickness skin served as control.

**Figure 2:**
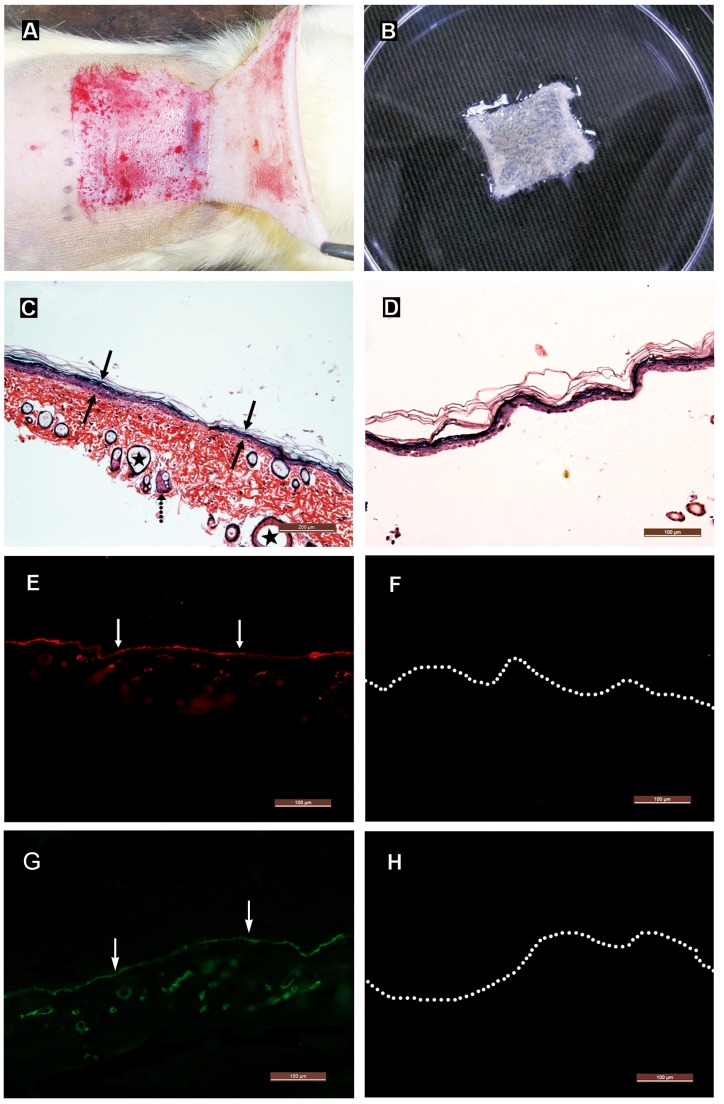
Histological characterization of epidermal sheet. (A) Gross appearance of the split-thickness skin. (B) Gross appearance of the epidermal sheet. It appears milk white, soft and elastic, and can be lifted and stretched by forceps. (C) H&E staining of the split-thickness skin. It is composed of an epidermis (solid arrows) and an underlying partial dermis, including some sebaceous glands (dotted arrow) and hair follicles (asterisks). Scale bar = 200 µm. (D) H&E staining of the epidermal sheet. It contains only an intact epidermis. Scale bar = 100 µm. (E–H) Immunohistochemical staining reveals that the split-thickness skin contains continuous distributions of collagen IV (arrows in E) and laminin (arrows in G) at the epidermal-dermal junction, and neither of them is detected in the epidermal sheet (F and H). Dotted lines indicate the location of basal keratinocytes. Scale bars = 100 µm.

**Figure 3:**
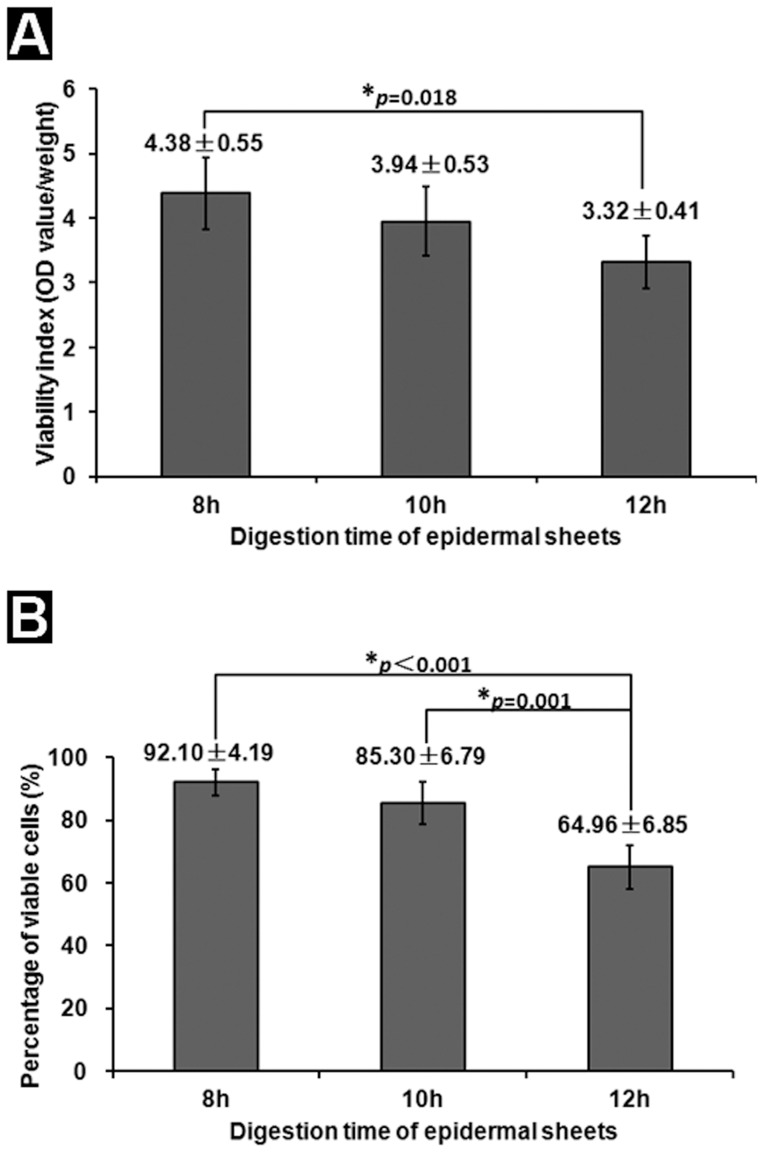
Tissue viability analysis. (A) Viability index of the epidermal sheet determined by CCK-8 assay. There is no significant difference in the viability index between the 8- and 10-h digestion groups, while the value of the 12-h digestion group is significantly lower than that of the 8-h digestion group. n = 5. (B) The percentage of viable cells detected by Hoe/PI staining. With the duration of Dispase II digestion prolonging, the percentage of viable cells in the epidermal sheet decreases gradually. n = 5.

### Observation of BM Structure

After being deparaffinized, rinsed and blocked with 4% bovine serum, the epidermal sheet sections were incubated with monoclonal antibodies against collagen IV (1∶150, BS2399, Bioworld, USA,) and laminin (1∶150, ab11575, ABcam, UK) at 4°C overnight. The primary antibodies were detected with TRITC and FITC conjugated secondary antibodies (1∶300, Santa Cruz, USA), respectively. Hoechst was used for nuclear counterstaining. The sections were examined under a fluorescent microscope (Leica 3000, Germany). The split-thickness skin served as control.

### Determination of Tissue Viability

#### CCK-8 assay

The epidermal sheet (1×1 cm) was placed in individual wells of a 24-well plate. 100 µl of CCK-8 solution was added to each well and incubated at 37°C for 4 h to form water dissoluble formazan. The formazan solution was then transferred to a 96-well plate to read the optical density (OD) value at 450 nm with a spectrophotometer (BioTek, USA). Then, the epidermal sheet was lyophilized and weighed (mg). The epidermal sheet incubated with 4% paraformaldehyde for 24 h served as control. The viability index of the epidermal sheet was calculated as its OD/weight. The results were averaged on five individual runs.

#### Hoe/PI staining

The epidermal sheet was spread flat over the bottom of a cell culture dish with its stratum corneum facing downward, and incubated with Hoechst 33342 solution (0.5 µl/ml in PBS) at 37°C for 10 min. Then PI solution (0.5 ml/ml in PBS) was added to the culture dish and continued with incubation for another 10 min. The sheet was finally rinsed with PBS twice and examined under a fluorescence microscope. PI could diffuse through the permeable membrane of dead cells and bind into their DNA, which could be detected by red fluorescent signal. Viable cells were able to take up Hoechst only and could be analyzed by blue fluorescence. Photographs were recorded with a digital camera (Canon, Japan). The percentage of viable cells was calculated using a computer-aided image analysis system (Image-Pro Plus, Media Cybernetics, USA) and averaged on nine random visual fields (200×) for each specimen. The results were averaged on five repeated experiments.

**Figure 4:**
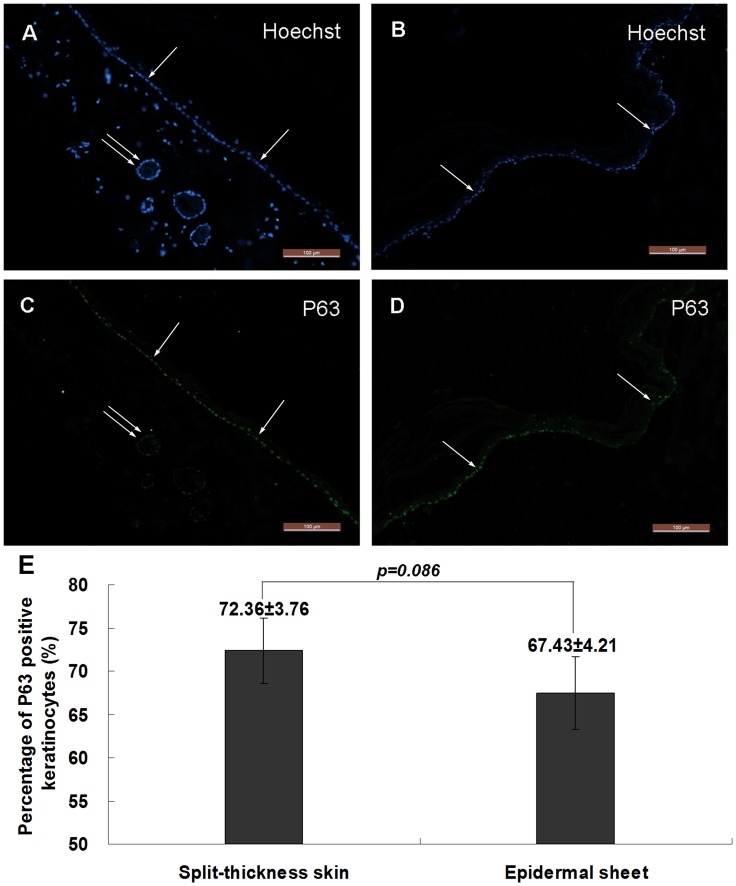
Cell proliferating ability analysis. (A–B) Hoechst staining shows the cellular structure of the split-thickness skin (A) and epidermal sheet (B). (C–D) Immunohistochemical staining reveals that most basal keratinocytes (single arrows) in the split-thickness skin (C) and epidermal sheet (D) are positive for P63. Cells around the follicular structures (double arrows) are also positive for P63. (E) The percentage of P63 positive keratinocytes in the epidermal sheet is not significantly different from that in the split-thickness skin (*p = *0.086, n = 5). Scale bars = 100 µm.

### Determination of Cell Proliferating Ability

Cell proliferating ability of the epidermal sheet was detected by immunohistochemical staining of P63 [17], [18]. Briefly, after being deparaffinized, rinsed and blocked with 4% bovine serum, the sections were incubated with monoclonal antibodies against P63 (1∶200, sc-8431, Santa Cruz, USA) at 4°C overnight. The primary antibody was detected with a FITC-conjugated secondary antibody (1∶300, Santa Cruz, USA), and Hoechst was used for nuclear counterstaining. The sections were observed under a fluorescent microscope and the split-thickness skin was used as control. The percentage of P63 positive keratinocytes was calculated using the Image-Pro Plus software and averaged on nine random visual fields (200×) for five individual specimens.

**Figure 5:**
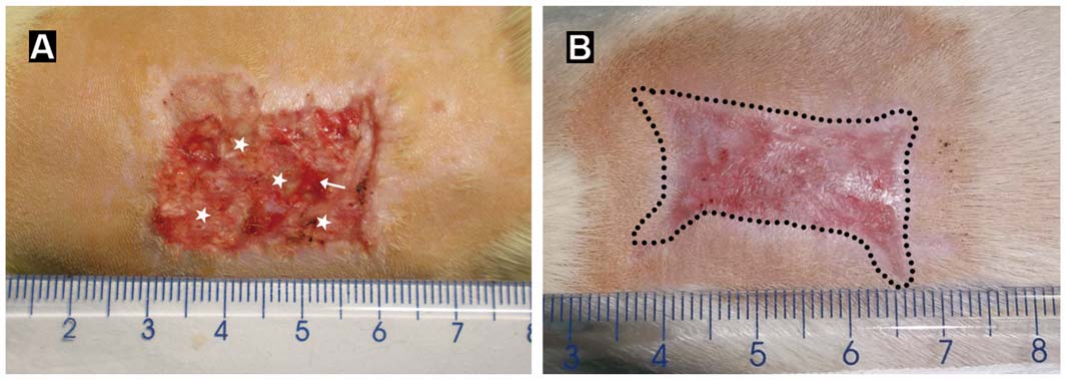
Wound healing process. (A) and (B) show gross appearance of the wound 2 and 3 weeks after co-transplantation of the epidermal sheet and ADM, respectively. The epidermal sheet survives and forms a new epidermis (asterisks in A) by week 2. The arrow indicates un-epithelialized area. By week 3, the wound is completely re-epithelialized. The newly generated epidermis becomes apparently thicker, and the healed wound surface is smooth with a mild degree of contraction (dotted lines in B).

**Figure 6:**
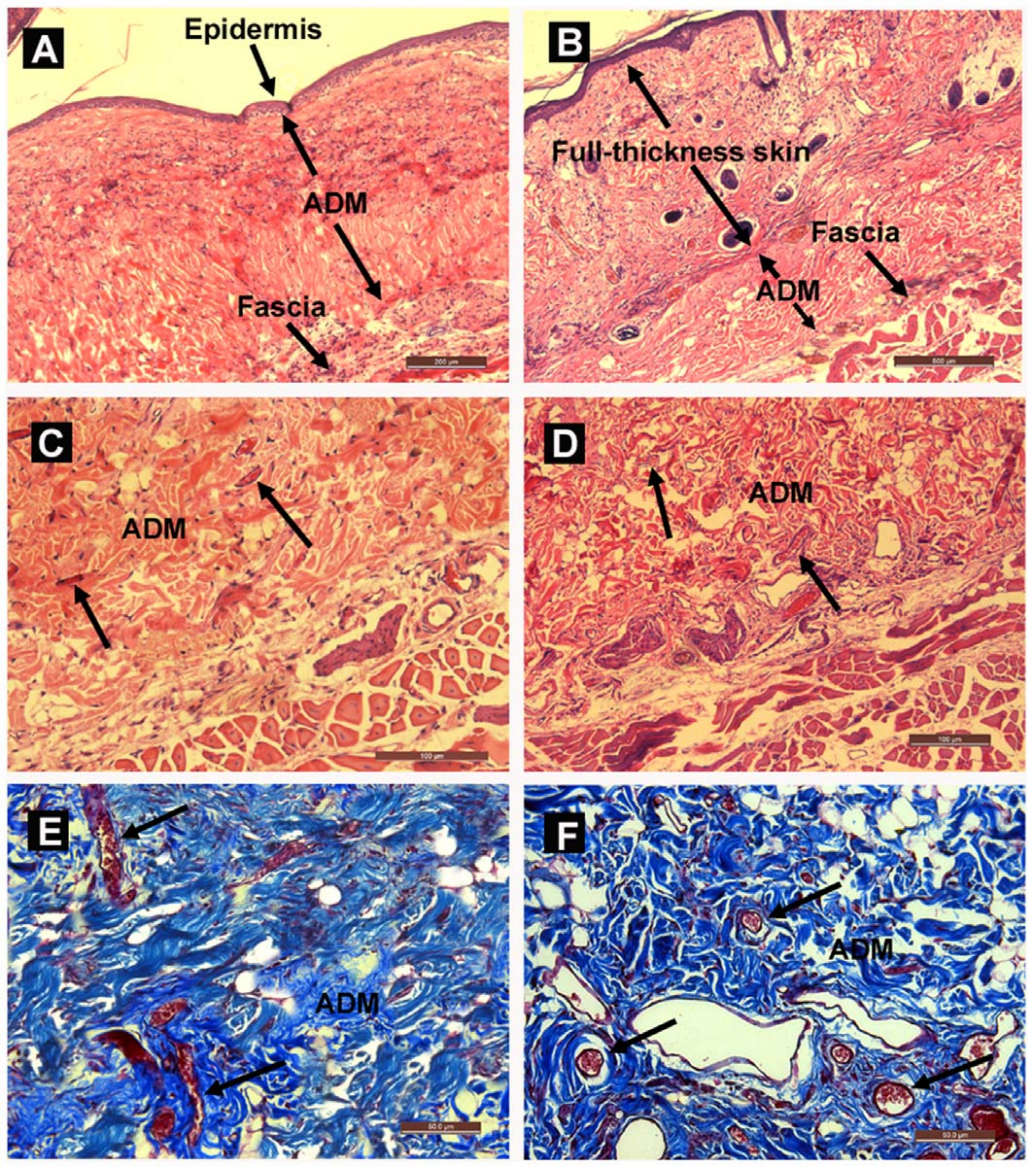
Histological analysis of the wound. (A) H&E staining of the wound 3 weeks after co-transplantation. The newly formed epidermis lies directly on the ADM surface. Scale bar = 200 µm. (B) H&E staining of the ADM 3 weeks after subcutaneous implantation. The ADM is laid between the host dermis and deep fascia. Scale bar = 500 µm. (C–D) H&E staining of the ADM-fascia junction area in the co-transplantation and subcutaneous implantation groups, respectively, indicating no apparent inflammatory response around the ADM, and fibroblasts and new blood vessels (arrows) have infiltrated into the ADM. Scale bars = 100 µm. (E–F) Masson’s trichrome staining of the ADM 3 weeks after co-transplantation (E) and subcutaneous implantation (F) show the broad presence of neo-capillaries (arrows) in both groups. Scale bars = 50 µm.

**Figure 7:**
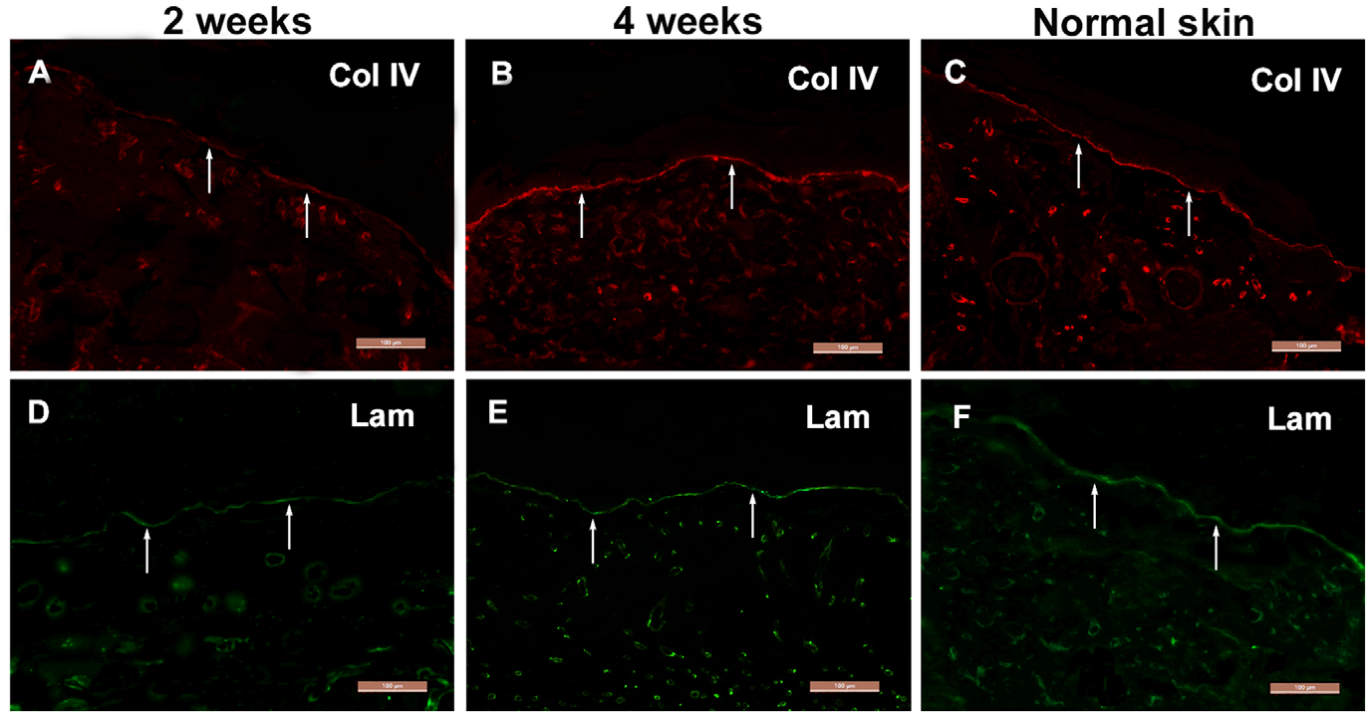
BM formation detected by immunohistochemistry. (A–C) Collagen IV and (D–F) laminin distribute intermittently at the epidermis-ADM junction of the wound 2 weeks after co-transplantation (arrows in A and D), and by week 4 their distributions have become continuous (arrows in B and E), similar to that in normal skin (arrows in C and F). Scale bars = 100 µm.

**Figure 8:**
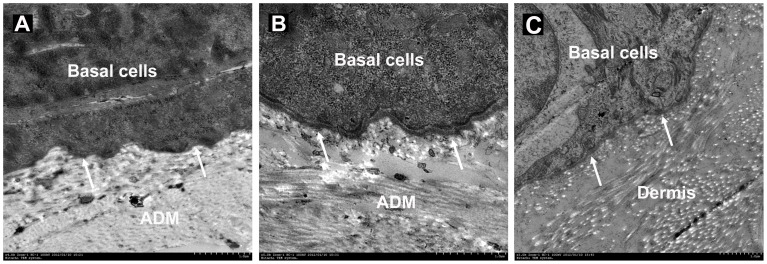
TEM. (A) Discontinuous lamina densa (arrows) exists at the epidermis-ADM junction of the wound 2 weeks after co-transplantation. (B) The laminin densa has become continuous and thick by week 4, and hemidesmosomes can also be observed between the basal keratinocytes and laminin densa. (C) The normal skin serves as control. Scale bars = 1 µm.

### Animal Transplantation Experiment

ADM was produced from the porcine skin as previously described with slight modifications [19]. Briefly, the porcine skin was subsequently treated with 0.25% trypsin (24 h) and 0.1% sodium dodecyl sulfate (24 h) to remove the epidermis and cell components in the matrix. The ADM was then further treated with Dispase II solution (2.4 U/ml) for 24 h at room temperature to get rid of the residual BM components, which was confirmed by the absence of BM-related protein collagen IV and laminin using immunohistochemical staining. The transplantation experiment was performed in 40 male SD rats (160–180 g), which were equally randomized into two groups.

#### Co-transplantation group (Figure 1)

The autologous epidermal sheet and ADM were co-transplanted to a full-thickness skin defect. The split-thickness rat skin was harvested as mentioned. The autologous epidermal sheet was obtained by incubating the split-thickness skin in Dispase II solution at an optimized condition. The remaining dermal tissue in the original area where the split-thickness skin was taken was removed to form a 3×3 cm full-thickness skin defect. The epidermal sheet obtained was placed over an ADM of the same size and transplanted compositely to the wound, fixed to the surrounding healthy skin with 4-0 silk sutures and covered with vaseline gauze. The wound was fixed with a bandage appropriately to avoid movement of the graft.

#### Control group

The ADM was subcutaneously implanted in the back. The rat was anesthetized, back shaven and sterilized with iodophor. A 3×3 cm full-thickness skin flap deep to the fascia of the back was dissected from one side. An ADM of the same size was implanted under the dissected skin flap with the papillary surface upwards and fixed to the surrounding tissue by intermittent sutures. Then the skin flap was returned to the original site and sutured.

### Observation of Wound Healing Process

The wound healing process was macroscopically observed daily after transplantation and photographed with a digital camera from a fixed distance. The Image-Pro Plus software was used to calculate the wound contraction rate using the formula: wound contraction rate  =  (original wound area - remaining wound area)/original wound area. Rats were sacrificed 1, 2, 3 and 4 weeks after transplantation, and wound specimens were collected for histological examination.

### Histological Evaluation

The skin and underlying subcutaneous tissue of the wounds were excised, fixed in 4% paraformaldehyde, dehydrated, paraffin-embedded and sliced into 5 µm sections. The sections were processed for routine H&E and Masson’s trichrome staining to observe the histological structure, according to the instructions provided by the manufacturers. H&E stained samples were examined for inflammation and vascularization by a board certified pathologist using a scale previously reported to evaluate the degree and type of inflammatory cells, as well as the presence of fibroblasts and vessels [20]. Masson’s staining showed blue collagen, red keratin and muscle fibers, and brown nuclei. BM-related protein collagen IV and laminin were detected by immunohistochemical staining using monoclonal antibodies against collagen IV and laminin, and visualized with secondary antibodies conjugated with TRITC and FITC fluorescence, respectively. The nuclei were stained by Hoechst dye.

### Transmission Electron Microscopy (TEM)

Part of the wound specimens were fixed with 2.5% glutaraldehyde in 0.1 M phosphate buffer (pH 7.4) for 24 h, washed with cacodylate buffer and post-fixed in 1% osmium tetroxide for 2 h. After washing in distilled water, the specimens were dehydrated and embedded in an Epon-resin mixture. The orientated blocks were cut for 0.5–1 µm sections using a Leica Ultracut S microtome and stained with 1% toluidine blue for light microscopic examination. Suitable parts of the sample were then selected for ultramicrotomy to obtain ultrathin sections (80 nm). The ultrathin sections were mounted on copper grids, double stained with uranyl acetate and lead citrate, and then examined with a transmission electron microscope (Hitachi H-7650, Japan) with 100 kV acceleration voltage [21].

### Statistical Analysis

Measurement data were consistent with normal distribution (Shapiro-Wilk method, *p*>0.10) and homogeneity of variance (Levene method, *p*>0.10). The results are expressed as mean ± standard deviation. Inter-group comparisons were performed using Student’s t-test (between two groups) or analysis of variance (between more than two groups, post hoc multiple comparisons were tested using Bonferroni method). Statistical analysis was performed using the SPSS16.0 software (SPSS Inc, Chicago, USA). *p*<0.05 was considered statistically significant.

## Results

### Histological Characteristics of Epidermal Sheet

The epidermal sheet was completely separated from the dermis after incubation of the split-thickness skin in Dispase II solution for 8 h or longer. The epidermal sheet appeared milk white, soft and elastic. It could be manipulated by forceps, was resistant to break on stretching, and easy to be sutured (Figure 2A–B).

H&E staining indicated that the split-thickness skin (0.3–0.5 mm in thickness) was composed of an intact epidermis and an underlying partial dermis, including some hair follicles and sebaceous glands (Figure 2C). The epidermal sheet (0.01–0.03 mm in thickness) only preserved an intact epidermis (Figure 2D). Immunohistochemical staining showed that collagen IV and laminin continuously existed at the epidermal-dermal junction in the split-thickness skin (Figure 2E and 2G), and there was no collagen IV and laminin component detectable in the epidermal sheet (Figure 2F and 2H), indicating that the BM and dermal components had been completely removed.

### The Epidermal Sheet Preserves High Viability

The mean viability index of the epidermal sheets in the 8- and 10-h digestion groups was 4.38±0.55 and 3.94±0.53, respectively, without significant difference between them (*p* = 0.583). The mean viability index of the epidermal sheet in the 12-h digestion group was 3.32±0.41, which was significantly lower than that of the 8-h digestion group (*p* = 0.018) (Figure 3A). The percentage of viable cells in the 8-, 10- and 12-h digestion groups was 92.10±4.19%, 85.30±6.79% and 64.96±6.85%, respectively (Figure 3B). The value of the 8-h digestion group was significantly higher than that of the 12-h digestion group (*p*<0.001). The viability of the epidermal sheet decreased gradually as the duration of Dispase II digestion prolonged. Based on these results, we decided to use the 8-h digestion epidermal sheet to continue with the following experiments.

### The Keratinocytes Maintain High Proliferating Ability

The results of anti-P63 immunohistochemical staining are shown in Figure 4. Most basal keratinocytes and cells around the follicular structures were positive for P63 in the split-thickness skin (Figure 4A and 4C). Most basal keratinocytes in the epidermal sheet were positive for P63 (Figure 4B and 4D). The percentage of P63 positive keratinocytes in the epidermal sheet was 67.43±4.21%, compared with 72.36±3.76% in the split-thickness skin, and there was no significant difference between them (Figure 4E, *p* = 0.086), indicating that 8-h Dispase II digestion did not significantly affect the proliferating ability of the keratinocytes.

### Gross Appearance of the Wound

The grafted epidermal sheet was well attached to the ADM 3 days after grafting, without falling-off or local necrosis seen, and the color of the epidermal sheet turned light pink gradually. 1 week after grafting, the epidermal sheet became reddish, indicting that it survived well. 2 weeks later, the original stratum corneum of the epidermal sheet fell off, and a new epidermis formed on the wound surface (Figure 5A). Then, the new epidermis became thicker continuously, and the wound was completely re-epithelialized 3 weeks after grafting. The healed wound looked smooth, with a wound contraction rate of 41.32±7.24% (Figure 5B). Of all the 20 rats in the co-transplantation group, only one epidermal sheet fell off and another one had local necrosis in the early period due to insecure fixation, and the other 18 epidermal sheets survived well and repaired the wounds.

### Histological Characteristics of the Wound

H&E staining showed that a stratified epidermis had formed and attached closely to the ADM 3 weeks after grafting, with little granulation tissue seen between them. The ADM was laid between the newly formed epidermis and the host deep fascia in the co-transplantation group, while it was placed between the host dermis and deep fascia in the subcutaneous implantation group (Figure 6A–B). There was no detectable fibrous capsule around the implanted ADM, and the ADM appeared healthy and had a dense cell population. The infiltrating cells appeared to be in fibroblastic morphology. There was also evidence of vascularization, as new blood vessels could be seen within the implanted ADM (Figure 6C–D). There were no notable differences in inflammatory cell infiltration and vascularization in the ADM between the co-transplantation and subcutaneous implantation groups (data not shown). The results of Masson’s trichrome staining further confirmed that the ADM in both groups was well vascularized with many neo-capillaries present (Figure 6E–F).

### BM Formation in the Wound

As shown in Figure 7, collagen IV and laminin existed intermittently at the epidermis and ADM junction of the wound 2 weeks after grafting (Figure 7A and 7D). On week 4, even and continuous collagen IV and laminin arrangements were observed at the junction (Figure 7B and 7E), similar to normal skin (Figure 7C and 7F). TEM demonstrated a hierarchical epidermis with the black and white bands of ADM collagen fibers seen clearly, with continuous distribution of condensed lamina densa at the basal keratinocytes and ADM junction, and hemidesmosome structures were observed between the basal keratinocytes and lamina densa (Figure 8).

## Discussion

A large number of new-type dermal substitutes have been developed and undergone animal experiments, and many studies reported promising results. However, only a few constructs have already been used in clinic [22]–[24]. One of the main reasons is that the animal models used are not capable of evaluating the safety and efficacy of these dermal substitutes reliably and comprehensively [25]. Safety and efficacy evaluation of a dermal substitute should include its biocompatibility, immunogenicity and the rate of revascularization, as well as its ability to promote new epidermis formation and dermal reconstruction.

Currently the overall strategy of dermal substitute evaluation consists of two parts: in vitro and in vivo [10]–[16]. The in vitro model is usually used to test the safety and toxicity of biological dermal substitutes, and as an initial screening to determine the feasibility of the dermal substitute. Then, the histocompatibility of the feasible dermal substitute as well as its effects on wound healing and dermal reconstruction have to be further investigated using the in vivo model. Most previous studies used the subcutaneous implantation model, in which the dermal substitute was subcutaneously embedded in the back or ear [11], [12], [26]. Although this model can effectively observe the inflammatory response and the revascularization rate after dermal substitute implantation, it cannot be used to directly observe its effect on the proliferation and differentiation of keratinocytes, neither as its effect on dermal reconstruction. Another important in vivo model is the wound healing model. By mimicking the clinical grafting technique, transplantation of autologous split-thickness skins compositely with dermal substitutes to repair full-thickness skin defects has been used to evaluate the dermal substitute [27]. However, since it is difficult to take the split-thickness skin of uniform thickness from small animals, the stability and reliability of this model are always questionable. In addition, because the split-thickness skin contains partial dermis and an intact BM, it is impossible to evaluate the actual effects of the dermal substitute on new epidermis and BM formation. There are also some other wound healing models [15], [16]. But as the procedure of one model is quite different from the other one, it is difficult to observe the efficacy of the dermal substitute accurately and stably.

In this study, we successfully used a Dispase II digestion method to obtain an epidermal sheet that contains an intact epidermis without any BM or dermal components. Dispase II decomposes collagen IV and fibronectin, and destroys the hemidesmosome structure at the epidermal-dermal junction, so as to isolate the epidermis from the dermis quickly and effectively [28]–[30]. However, the viability of the epidermal sheet is to some extent correlated with the duration of Dispase II digestion. The tissue viability decreases as the duration of digestion prolongs. It was found in our study that 8-h Dispase II digestion was able to separate the epidermis from the dermis completely, and the epidermal sheet obtained not only preserved an intact epidermis without any dermal or BM structures, but also maintained high tissue viability. P63 is a nuclear transcription factor expressed in undifferentiated proliferating cells and is critically important for skin and appendage development and maintenance [17], [18]. The results of our immunohistochemical staining showed that the percentage of P63 positive keratinocytes in the epidermal sheets was similar with normal skin, indicating that the keratinocytes maintained good proliferating ability. With necessary thickness and toughness, the epidermal sheet is also suitable for surgical manipulations.

Subsequently, we co-transplanted the epidermal sheet with ADM to repair autologous full-thickness skin defect, and observed the effects of the ADM on wound healing and dermal reconstruction. Previous studies showed that epidermal sheets cultured in vitro had a low survival rate when transplanted onto a non-vascularized dermal substitute [2]. It was unexpectedly found in our study that the grafted epidermal sheet survived well and completely re-covered the wounds within 3 weeks. We suppose that the presence of stratum corneum within the epidermal sheet may have played an important role in keeping the graft in a moist environment, which is critical for the survival and proliferation of the keratinocytes. The thin ADM allowed nutrients to permeate during early period and was well vascularized within 3 weeks after grafting, allowing for quick re-epithelialization and dermal reconstruction. These results are consistent with earlier studies [31]–[33].

Some studies have demonstrated that the BM structure could be preserved in ADM, and this pre-existing BM was beneficial to the adhesion and proliferation of keratinocytes and promoted new BM formation [10], [33], [34]. However, these conclusions were based on the results of in vitro studies, and no studies available have used effective animal models to determine whether this pre-existing BM could promote new epidermis and BM formation during the wound-healing process. In the present study, by co-transplanting the autologous epidermal sheet with ADM (without BM) to a full-thickness skin defect, we were able to directly observe the effects of ADM on new epidermis and BM formation during the wound-healing process. Our model can be further used to observe the effects of the pre-existing BM in ADM on epidermis and BM formation by grafting the autologous epidermal sheet with ADM (with BM) to full-thickness skin defects in the future.

This novel animal model is capable of investigating the biocompatibility of the dermal substitute as in subcutaneous implantation model, and capable of examining the effects of the dermal substitute on new epidermis and BM formation. Therefore, it is a useful and supplementary model to the current in vivo evaluation system to investigate the in vivo behavior of the dermal substitute. However, comparison experiments between this model and the other wound healing models still need to be performed in the future to evaluate its reliability and effectiveness.

### Conclusion

In this study we have constructed a novel dermal substitute evaluation model by co-transplanting dermal substitutes with separated autologous epidermal sheets to repair full-thickness skin defects. This model can be used not only to observe the biocompatibility of dermal substitutes, but also to evaluate their effects on new epidermis and BM formation during the wound healing process. It may prove to be a convenient and reliable model for evaluating the safety and efficacy of dermal substitutes.
